# Differential inhibition of LINE1 and LINE2 retrotransposition by vertebrate AID/APOBEC proteins

**DOI:** 10.1186/1742-4690-10-156

**Published:** 2013-12-17

**Authors:** Nataša Lindič, Maruška Budič, Toni Petan, Binyamin A Knisbacher, Erez Y Levanon, Nika Lovšin

**Affiliations:** 1 Department of Chemistry and Biochemistry, Chair of Biochemistry, Faculty of Chemistry and Chemical Technology, University of Ljubljana, SI-1000, Ljubljana, Slovenia; 2 Department of Molecular and Biomedical Sciences, Jožef Stefan Institute, SI-1000, Ljubljana, Slovenia; 3 The Mina & Everard Goodman Faculty of Life Sciences, Bar-Ilan University, Ramat Gan 52900, Israel

**Keywords:** G to A hypermutation, AID, APOBEC3, ORF1p, Lizard APOBEC1, Zebrafish LINE2, LINE1, Zebrafish APOBEC2, Cytidine deaminase, Retrotransposon

## Abstract

**Background:**

The role of AID/APOBEC proteins in the mammalian immune response against retroviruses and retrotransposons is well established. G to A hypermutations, the hallmark of their cytidine deaminase activity, are present in several mammalian retrotransposons. However, the role of AID/APOBEC proteins in non-mammalian retroelement restriction is not completely understood.

**Results:**

Here we provide the first evidence of anti-retroelement activity of a reptilian APOBEC protein. The green anole lizard A1 protein displayed potent DNA mutator activity and inhibited *ex vivo* retrotransposition of LINE1 and LINE2 ORF1 protein encoding elements, displaying a mechanism of action similar to that of the human A1 protein. In contrast, the human A3 proteins did not require ORF1 protein to inhibit LINE retrotransposition, suggesting a differential mechanism of anti-LINE action of A1 proteins, which emerged in amniotes, and A3 proteins, exclusive to placental mammals. In accordance, genomic analyses demonstrate differential G to A DNA editing of LINE retrotransposons in the lizard genome, which is also the first evidence for G to A DNA editing in non-mammalian genomes.

**Conclusion:**

Our data suggest that vertebrate APOBEC proteins differentially inhibit the retrotransposition of LINE elements and that the anti-retroelement activity of APOBEC proteins predates mammals.

## Background

**Figure 1:**
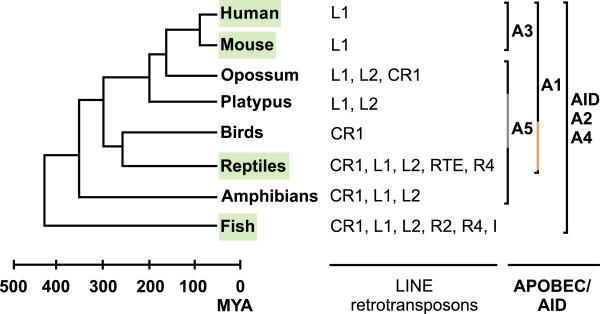
**Phylogenetic relationships between the AID/APOBEC proteins and LINE retrotransposon families in various vertebrates.** The scheme shows different clusters of AID/APOBEC family members identified in various vertebrates and LINE retrotransposons present in their genomes. The genomes and/or AID/APOBEC proteins analyzed in this study are marked green, and the absence of A5 protein in platypus and birds is marked with a grey line. The presence of the only A1 homologs identified in non-mammalian genomes so far, green anole lizard and zebra finch A1 proteins, is marked with an orange line. Data was derived from previous studies [18,20-34]. One unit on the timeline represents 100 million years. MYA, millions years ago.

Retrotransposons constitute almost half of the human genome and are considered to be one of the major driving forces in the evolution of eukaryotic genomes [1]. They are classified into two major types, long terminal repeat (LTR) retrotransposons, which include retroviruses, and non-LTR retrotransposons [2]. The non-LTR retrotransposon LINE1 (L1) and LINE2 (L2) clades, which are widespread among vertebrates, differ in two important structural and functional characteristics. First, the L1 retrotransposon carries two open reading frames (ORF) encoding ORF1p, an RNA binding protein [3-7], and ORF2p, a polyprotein with endonuclease and reverse transcriptase activity [8-10]. In contrast, the L2 retrotransposons can encode either one (ORF2p) or two ORF proteins [11,12], ORF1p being expendable for retrotransposition in cultured cells [13]. Second, unlike the L1 reverse transcriptase that can mobilize other RNA species [14-16], the L2 enzyme is specific for its own 3′ UTR [12,17]. Furthermore, while both L1 and L2 elements are present in fish, amphibians and reptiles [18-20], only the L1 retrotransposon clade has greatly expanded in mammals [21], reaching 17% of the human genome [22]. In contrast, the L2 retrotransposons are inactive in placental mammals, with only highly defective copies present in the human genome [18,22]. In fact, a massive reduction in the diversity of active LINE retrotransposon families occurred during the evolution of tetrapod genomes (summarized in Figure 1). This ancient conflict between the retroelements and their hosts has driven the evolution of many host defense systems (reviewed in [2]), one of them being the AID/APOBEC proteins.

AID/APOBEC protein family members are cytidine deaminases (CDA) that convert cytidine to uridine in DNA and RNA [23]. They emerged at the origin of vertebrates and have spread throughout the vertebrate lineage (summarized in Figure 1). The early ancestors of AID/APOBEC proteins have been identified in jawless and cartilaginous fish and include the activation-induced deaminase (AID), important for antibody diversification, and the APOBEC2 (A2) protein, involved in active demethylation and muscle development [35-38]. In amphibians, APOBEC4 (A4) and APOBEC5 (A5) proteins emerged, but their functions are yet to be identified [23,24,26]. During the tetrapod evolution, duplications of AID genes led to the development of APOBEC1 (A1) [24,35,39-41]. In placental mammals the APOBEC3 (A3) genes evolved and greatly expanded. While one gene is present in mice, there are 7 human A3 genes: A3A, A3B, A3C, A3D, A3F, A3G and A3H [24,29-31,35,42,43]. A3 proteins inhibit various viruses by introducing C to U mutations in viral cDNA intermediates leading to G to A hypermutations and viral inhibition (reviewed in [44]). Alternatively, A3 proteins may inhibit retroviruses and LINE elements by inhibiting reverse transcription [45-48]. A3 proteins have been shown to inhibit many LTR and non-LTR retrotransposons [49-54], but also various mammalian A1 and pre-mammalian AID proteins exhibit an activity against the human L1 (hL1) retrotransposon [55,56]. Moreover, the effects of AID/APOBEC CDA activity have been identified in the sequence of several mammalian LTR and non-LTR retrotransposons [57,58], but not yet in non-mammalian genomes, which lack A3 proteins. Recently, it has been shown that the lizard A1 protein possesses DNA deaminase activity [24], which is consistent with the hypothesis that the original function of AID/APOBEC family of enzymes is retroelement restriction, rather than antibody diversification [59].

To examine the hypothesis that retroelement restriction by APOBEC proteins predates mammals, we performed comparative analyses of the impact of currently available vertebrate APOBEC proteins on the retrotransposition of vertebrate LINE elements and performed genome-wide DNA editing analyses of vertebrate genomes. We found G to A DNA hypermutations in green anole lizard retrotransposons, which is the first evidence of retrotransposon editing in non-mammalian vertebrate genomes. The green anole lizard A1 protein displayed potent DNA mutator and anti-retroelement activity, which paralleled that of the human A1 protein. Our results further reveal a differential mechanism of action of A1 and A3 proteins against LINE retrotransposons.

## Results

### Lizard A1 protein inhibits hL1 and ZfL2-1, but not ZfL2-2, retrotransposons

**Figure 2:**
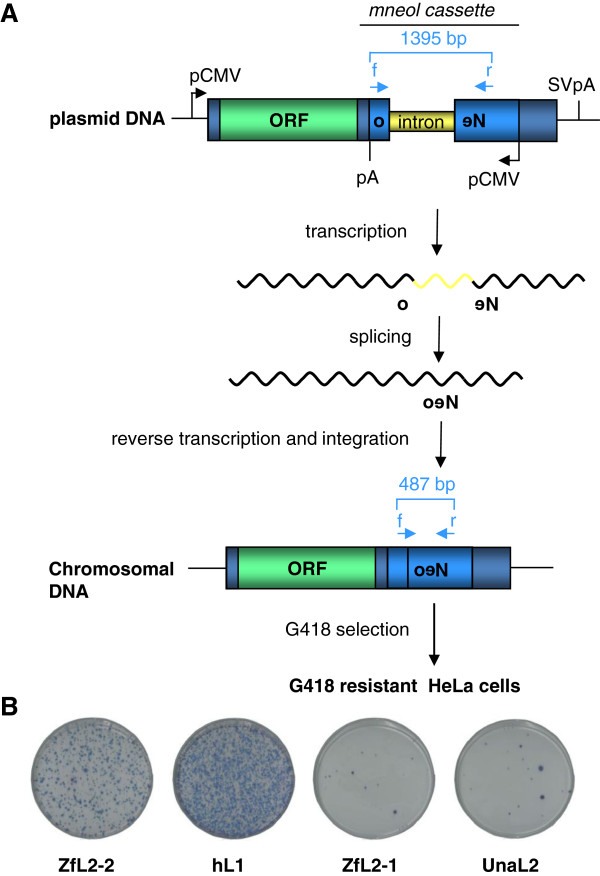
**Overview of the *neo*-based retrotransposition assay in HeLa cells (adapted from**[60]**) and basal retrotransposition levels. (A)** HeLa cells were transfected with plasmids encoding the *neo*-marked zebrafish L2-2 (ZfL2-2) [11], human L1 (hL1) (specifically L1.2) [60], zebrafish L2-1 (ZfL2-1) [11], or eel L2 (UnaL2) [12] retrotransposons. The neomycin resistance cassette (*neo*), inserted within the 3′ UTR region of each tested retrotransposon in the opposite direction, is interrupted by an intron from the human γ-globin gene in the sense orientation. Transcription of the retrotransposon DNA containing the cassette, splicing of the γ-globin intron, reverse transcription and integration in the genome are required for expression of the *neo* gene. The number of G418 resistant colonies obtained after 12 days of selection is proportional to the number of successful retrotransposition events. The position of primers used to discriminate between the intronless and unspliced retrotransposon copies and the sizes of PCR products are indicated. **(B)** Representative experimental results obtained in the absence of APOBEC proteins after selection of neomycin resistant colonies for 12 days. The approximate basal retrotransposition rates of each tested retrotransposon, expressed, in average number of colonies per plate, were: 4958 (split into 5 plates for counting) for hL1, 782 for ZfL2-2, 54 for ZfL2-1 and 101 for UnaL2 retroelements. pCMV, cytomegalovirus promoter; SVpA, SV40 poly A signal; f, neo437s primer; r, neo1808as primer. To rule out the possibility that the observed LINE inhibition was due to a non-specific toxicity of the tested AID/APOBEC proteins, the number of G418 resistant colonies obtained upon co-transfection of HeLa cells with an AID/APOBEC encoding and a *neo*-expressing pcDNA3.1 plasmid was determined (Additional file 1: Figure S1) as described in [53] and [61].

The CDA activity of the zebrafish AID protein and its weak but present anti-retroelement activity, observed against the hL1 element [56], suggest that zebrafish APOBEC proteins could also act as inhibitors of retrotransposition, in particular against zebrafish elements. To test this idea, a neomycin resistance gene (*neo)*-based retrotransposition assay (Figure 2) was performed. Human A1 and A3A proteins, potent hL1 inhibitors [49,51,55], were used as positive controls. Surprisingly, the zebrafish A2a, A2b and AID proteins had no effect on the retrotransposition of the zebrafish L2-1 (ZfL2-1) or L2-2 (ZfL2-2) elements, with A2a and AID displaying only a very weak inhibitory effect against the hL1 element (Additional file 1: Figure S2). The level of zebrafish AID-induced hL1 inhibition was similar to that previously detected using a GFP-based retrotransposition assay in HEK293 cells [56].

**Figure 3:**
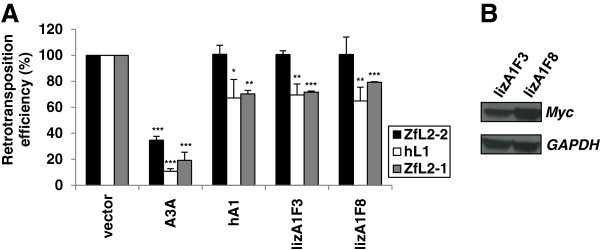
**Lizard and human A1 proteins inhibit ORF1p encoding hL1 and ZfL2-1, but not ZfL2-2 retrotransposition. (A)** HeLa cells were co-transfected with ZfL2-1, ZfL2-2 or hL1 retrotransposon plasmids and effector plasmids encoding human A3A, human A1 (hA1), or lizard A1a protein variants F3 and F8 (lizA1F3 and lizA1F8). Relative retrotransposition efficiency was determined by counting the fixed and stained neomycin resistant colonies, formed after 12 days of G418 selection, and calculated by setting the value obtained for control cells co-transfected with retrotransposon plasmid and an empty vector at 100%. Data are the means ± SD of three independent experiments. ***P < 0.001, **P < 0.01, *P < 0.05, *t*-test. **(B)** The expression of Myc epitope-tagged lizard proteins in mammalian cells was confirmed in the HEK293T cell model by immunoblotting. GAPDH expression levels were used as a loading control.

Given that the recently identified lizard A1 protein displays DNA mutator activity [24], we next asked whether this non-mammalian APOBEC protein exhibits anti-retroelement activity. Surprisingly, we found that the two natural isoforms of the lizard A1a protein (lizA1F3 and lizA1F8) decrease the retrotransposition rates of hL1 to 70%, thus displaying an inhibition potency similar to that observed for the human A1 protein (Figure 3). In contrast, no inhibitory activity against the ZfL2-2 element was detected for the lizard A1 proteins, but they efficiently inhibited the ZfL2-1 retrotransposon, displaying an effect comparable with that exhibited against the hL1 retrotransposon. The tested proteins were not cytotoxic (Additional file 1: Figure S1) and had no effect on the transfected plasmid DNA (Additional file 1: Figure S5) or the expression from it (Additional file 1: Figure S6). Since one of the major differences between the ZfL2-1 and ZfL2-2 retrotransposons is the absence of ORF1p in the latter [11], our results suggest that the presence of ORF1p contributes to the inhibition of LINE retrotransposons by A1 proteins. Our results clearly suggest a role for lizard A1 proteins in anti-retroelement defense.

### Zebrafish A2b and lizard A1 proteins exhibit DNA mutator activity

**Figure 4:**
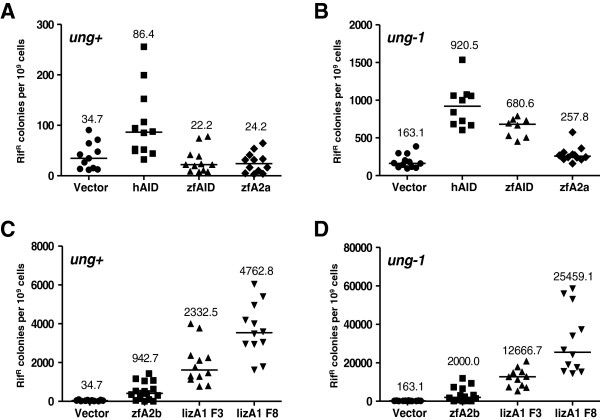
**DNA editing activities of various AID and APOBEC proteins determined with the *E. coli *mutator assay. (A, C)** Frequencies of rifampicin resistant (Rif^R^) colonies in KL16 (*ung+*) *E. coli* cultures carrying human AID (hAID), zebrafish AID (zfAID), zebrafish A2a (zfA2a), zebrafish A2b (zfA2b) and lizard A1 variants F3 (lizA1F3) and F8 (lizA1F8) expression plasmids or the vector as control. **(B, D)** Mutation frequencies of BW310 (*ung-1*) *E. coli* cultures expressing AID/APOBEC proteins or a vector control as in A and C, but note the larger scale on the y-axis. Each point represents the mutation frequency of an independent overnight culture. Median mutation frequencies are indicated.

To confirm the recently reported DNA mutator activity of the lizard A1 protein [24] and to examine the possibility that the zebrafish A2 proteins also possess CDA activity, we employed the bacterial rifampicin fluctuation assay. Mutations in the bacterial *rpo* gene induced by an active DNA deaminase transiently expressed in bacteria were quantified by measuring the number of rifampicin resistant (Rif^R^) colonies [62-64]. Expression of the anole lizard A1 F3 protein led to a 77-fold increase in mutational frequency, while the A1 F8 protein caused a 156-fold increase over control values obtained with an empty plasmid (Figure 4D). Zebrafish and human AID proteins increased the mutational frequency 5.6- and 4-fold, respectively (Figure 4B), which accords with previous reports [56,62]. Whereas the expression of the zebrafish A2b protein in *E. coli* cells resulted in a 12-fold higher mutational frequency (Figure 4D), the zebrafish A2a protein had a negligible effect on the number of Rif^R^ colonies (Figure 4B). For all proteins, the mutator activity was enhanced in the absence of uracil-DNA glycosylase (UDG) (compare Figure 4A with B, and C with D). Our results demonstrate that the zebrafish A2b and lizard A1 proteins are potent DNA mutators.

### Human A3 proteins inhibit ZfL2-2 retrotransposition

**Figure 5:**
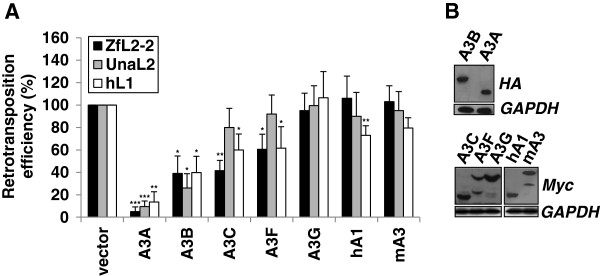
**Human A3A, 3B, 3C and 3F proteins restrict ZfL2-2 retrotransposition. (A)** Inhibitory effect of mouse and human A3 proteins on retrotransposition of hL1, ZfL2-2 and UnaL2 retrotransposons. HeLa cells were co-transfected with 1 μg of the indicated APOBEC effector plasmid and 1 μg of retrotransposon target plasmid. Relative retrotransposition efficiency was determined by counting the fixed and stained neomycin resistant colonies, formed after 12 days of G418 selection (Additional file 1: Figure S3), and calculated by setting the value obtained for control cells co-transfected with retrotransposon plasmid and an empty vector at 100%. Data are the means ± SD of at least three independent experiments. ***P < 0.001, **P < 0.01, *P < 0.05, *t*-test. **(B)** The expression of HA, Myc, or V5 epitope-tagged proteins tested in **(A)** was confirmed in the HEK293T model by immunoblotting. GAPDH protein levels were used as a loading control.

Based on the activity of the human A3A protein against ZfL2-2 and ZfL2-1 (Figure 3 and Additional file 1: Figure S2), we hypothesized that other human A3 proteins can also inhibit L2 elements such as the zebrafish ZfL2-2 and eel L2 (UnaL2) retrotransposons. Indeed, both fish L2 retrotransposons were most potently inhibited by the A3A and A3B proteins, that decreased the retrotransposition efficiency of the ZfL2-2 retrotransposon to 5% and 35%, and of the UnaL2 retrotransposon to 10% and 25%, respectively (Figure 5 and Additional file 1: Figure S3). The human A3C and A3F proteins also inhibited ZfL2-2 retrotransposition, but to a lesser extent, and did not affect UnaL2 retrotransposition significantly. The human A3G and mouse A3 proteins (Figure 5), as well as the human A2, AID, A3D and A3H-L (haplotype I) proteins (Additional file 1: Figure S4) had no significant inhibitory activity against the ZfL2-2 retrotransposon. Notably, the inhibitory potencies of human A3 proteins against the fish L2 retrotransposons were comparable to those against the hL1 retrotransposon, as observed by us (Figure 5, and Additional file 1: Figures S2–S4) and others [49,51,53,65,66]. Any significant degradation of transfected ZfL2-2 plasmid DNA by A3 proteins in the retrotransposition assays, as reported for the human A3A protein and foreign DNA [67], was excluded using quantitative real-time PCR (qPCR) analysis (Additional file 1: Figure S5). Additionally, the A3A and A3B proteins were only slightly toxic to the cells during the long period of selection required for the retrotransposition assay (Additional file 1: Figure S1), and had no effect on plasmid transcription (Additional file 1: Figure S6), thus confirming *bona fide* retrotransposon restriction mechanisms. Altogether, we demonstrate that human A3A and A3B proteins are potent inhibitors of ZfL2-2 and UnaL2 retrotransposition in HeLa cells, and that the human A3 proteins inhibit L1 and L2 retrotransposons with a similar potency.

### Intact A3A active site is required for ZfL2-2 inhibition

**Figure 6:**
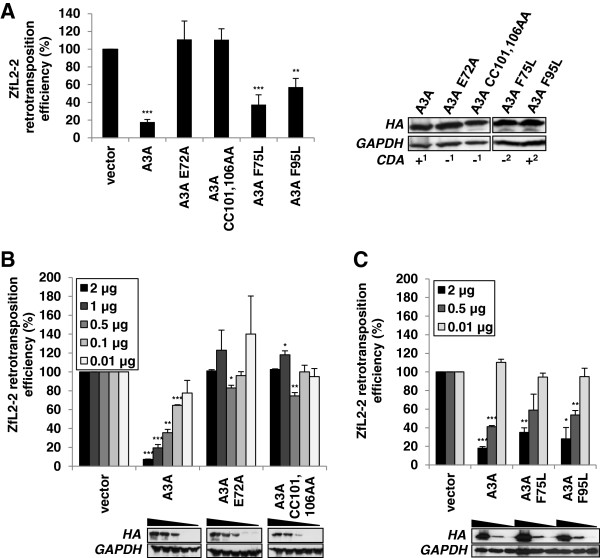
**Intact catalytic site is required for ZfL2-2 inhibition by the human A3A protein.** HeLa cells were co-transfected with 1 μg of target ZfL2-2 plasmid and **(A)** 1 μg of plasmid encoding the A3A effector or its E72A, CC101,106AA, F75L and F95L mutants, **(B)** 2, 1, 0.5, 0.1 and 0.01 μg of A3A and its catalytic site mutants E72A and CC101,106AA, or **(C)** 2, 0.5 and 0.1 μg of A3A and its F75L and F95L mutants. Neomycin resistant colonies, formed after G418 selection, were fixed, stained and counted, and relative retrotransposition efficiency was calculated by setting the value obtained for cells co-transfected with the retrotransposon plasmid and an empty vector at 100%. The reported cytidine deaminase activity (CDA), or lack thereof, of each protein is designated with + or – (1, ref. [49]; 2, ref. [68]). Western blotting was performed using extracts from 293T cells transiently expressing HA epitope-tagged proteins. GAPDH protein levels were used as loading controls. Data are the means ± SD of at least three independent experiments. ***P < 0.001, **P < 0.01, *P < 0.05, *t*-test.

Since the human A3 proteins inhibited both L1 and L2 elements, we hypothesized that there are common structural features of A3 proteins required for their inhibition. To determine if the integrity of the active site residues of the single domain A3A protein is required for ZfL2-2 inhibition, as shown previously for hL1 inhibition [49,51,53], the retrotransposition assay was performed with two active site A3A mutants (E72A and CC101,106AA) that lack CDA activity [49,51]. The mutations completely abolished the inhibitory activity of the A3A protein against the ZfL2-2 retrotransposon (Figure 6A), which was confirmed in a dose-dependent experiment (Figure 6B). To further examine the role of A3A enzymatic activity in ZfL2-2 inhibition, we mutated two other structurally conserved residues F75 and F95, essential for deaminase activity in the A1 protein, but differentially affecting the CDA activity of A3A [68,69]. Namely, while only the A3A F95L mutant displayed deaminase activity *in vitro*, both F75L and F95L mutants retained their ability to restrict adeno-associated-virus replication [68]. Interestingly, both mutants decreased the retrotransposition rates of the ZfL2-2 retrotransposon to 40% (Figures 6A and C). Although the lack of deaminase activity of the F75L mutant [68] awaits further confirmation in cellular *ex vivo* assays, this indicates that A3A enzymatic activity is dispensable for ZfL2-2 inhibition, which is in accordance with a recent report showing that the determinants of A3A CDA activity and anti-hL1 activity are not the same [70]. Our results thus suggest that while an intact active site of the A3A protein is required for successful inhibition of ZfL2-2, enzymatic activity may not be necessary.

### The integrity of at least one A3B domain is required for ZfL2-2 inhibition

**Figure 7:**
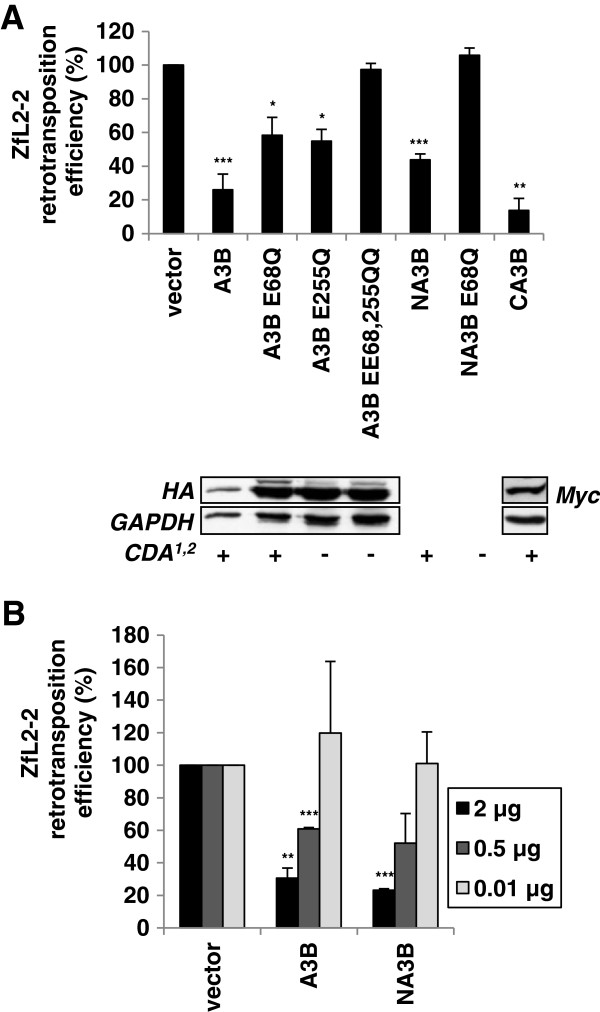
**At least one A3B domain with catalytic site integrity is necessary for ZfL2-2 inhibition.** HeLa cells were co-transfected with 1 μg of target ZfL2-2 plasmid and **(A)** 1 μg of effector plasmid coding for the wild-type A3B protein or its mutants, or **(B)** 2, 0.5 and 0.01 μg of plasmids encoding the A3B protein and its C-terminal deletion mutant, NA3B. Relative retrotransposition efficiency was determined by counting the fixed and stained neomycin resistant colonies, formed after 12 days of G418 selection, and calculated by setting the value obtained for control cells co-transfected with retrotransposon plasmid and an empty vector at 100%. The reported cytidine deaminase activity (CDA), or lack thereof, of each protein is designated with + or – (1, ref. [51]; 2, ref. [71]). Western blotting was performed using extracts from 293T cells transiently expressing HA and Myc epitope-tagged proteins. GAPDH protein levels were used as loading controls. Data are the means ± SD of at least three independent experiments. ***P < 0.001, **P < 0.01, *P < 0.05, *t*-test.

To investigate whether the enzymatic activity of the A3B protein is important for the inhibition of ZfL2-2 retrotransposition, as previously observed for hL1 inhibition [51,65], we employed several A3B mutants. The A3B protein possesses two catalytically active domains with a CDA consensus site, the N-terminal domain (NA3B) with nucleic acid binding function, and the C-terminal domain (CA3B) responsible for enzymatic activity [71]. Interestingly, the single domain mutants CA3B and NA3B inhibited ZfL2-2 retrotransposition with a potency similar to that of the wild-type A3B protein (Figure 7), suggesting that either domain is sufficient for the inhibition. The inhibitory effect of the NA3B mutant, confirmed by a dose-dependent retrotransposition assay (Figure 7B), was completely alleviated when the glutamate residue in the consensus site was mutated to glutamine (NA3B E68Q) (Figure 7A). Furthermore, the A3B E68Q and A3B E255Q mutants, with N- and C-terminal domain-specific impairment of CDA activity [71], respectively, only partially restricted ZfL2-2 retrotransposition, while the double mutant A3B EE68, 255QQ completely lost the inhibitory activity, strongly suggesting that the enzymatic activity of at least one A3B domain is required for ZfL2-2 restriction. These results clearly show that the integrity of one A3B domain is sufficient and suggest that A3B CDA activity is necessary for the ZfL2-2 inhibition.

### A3, but not A1 and A2, proteins decrease the amount of novel LINE DNA copies

**Figure 8:**
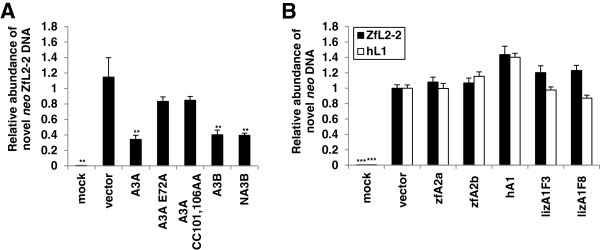
**A3A and A3B, but not A1 or A2, proteins decrease the amount of novel ZfL2-2 DNA. (A, B)** Total cellular DNA was extracted from HeLa cells after 4 days of hygromycin selection following transfection with plasmids encoding ZfL2-2 or hL1 retrotransposons and the indicated APOBEC effector plasmids (1 μg of each). Isolated DNA was subjected to qPCR for specific quantification of intronless amplicons from the spliced *neo* cassette corresponding to both integrated and *de novo* synthesized retrotransposon DNA. Values obtained with DNA from cells transfected with an empty vector were set as 1. Histogram bars represent the means ± SD of two independent experiments. Mock controls represent untransfected cells. ***P < 0.001, **P < 0.01, *P < 0.05, *t*-test.

The human A3A and A3B proteins inhibit hL1 retrotransposition by decreasing the number of novel hL1 DNA [54,55,65,66,69]. To determine whether APOBEC-induced ZfL2-2 inhibition is due to a similar mechanism, the effects of A3A and A3B proteins and their mutants on the amount of novel ZfL2-2 DNA copies were determined by qPCR (Figure 8). The specificity of the primers spanning the exon-exon junction, targeting spliced and reverse-transcribed *neo* fragments, thus enabling a detection of both newly integrated ZfL2-2 genomic copies and novel ZfL2-2 cDNA, was confirmed (Additional file 1: Figure S8). Our results demonstrate that the human A3A and A3B proteins, as well as the single domain NA3B mutant, that all potently inhibit ZfL2-2 retrotransposition (Figures 5, 6 and 7), also decrease the level of novel ZfL2-2 DNA (Figure 8A). The enzymatically inactive A3A mutants, that were ineffective in the ZfL2-2 retrotransposition assay (Figure 6), did not significantly affect the amount of novel ZfL2-2 DNA copies (Figure 8A), confirming that the anti-retroelement activity and the decrease in ZfL2-2 DNA are a consequence of wild type A3A protein expression. Even though foreign DNA degradation by A3 proteins has been suggested previously [67], the effect observed on the level of cDNA was due neither to significant plasmid degradation (Additional file 1: Figure S5A) nor to a decrease in expression from the plasmid (Additional file 1: Figure S6A). This is in agreement with the dominant effect of the A3A protein on the inhibition of hL1 retrotransposition rather than on hL1 plasmid degradation reported previously [69]. Since the impact of A3 proteins on the level of novel retrotransposon DNA (Figure 8) corresponds to the results of the retrotransposition assay (Figures 5, 6 and 7), we suggest that the inhibition of ZfL2-2 retrotransposon occurs at the reverse transcription and/or at the integration step.

Interestingly, despite their anti-retroelement activity against hL1 (Figure 3), the lizard and human A1 proteins did not change significantly the level of novel hL1 or ZfL2-2 DNA (Figure 8B). The possibility of A1 proteins affecting the level of the transfected plasmid or the level of expressed retrotransposon RNA was excluded by performing qPCR analyses (Additional file 1: Figures S5B and S6B). Our results thus suggest that the inhibition of hL1 retrotransposition by A1 proteins does not include significant alterations in the amount of novel retrotransposon DNA, thus implying that lizard and human A1 proteins do not interfere with the stability or the synthesis of the retrotransposon DNA.

### Novel ZfL2-2 and hL1 DNA copies are not hypermutated

We next asked whether any mutations might have occurred in the novel spliced ZfL2-2 and hL1 DNA copies in the presence of zebrafish, lizard, mouse or human APOBEC proteins. The overall mutation rates were very low and there was no apparent increase in G to A mutations (Additional file 1: Figure S7), indicating that neither of the tested AID/APOBEC proteins edits the novel retrotransposon DNA during the course of reverse transcription or integration. This is in accordance with previous studies showing that the inhibition of hL1 by human AID/APOBEC proteins occurs in the absence of hypermutations [49,51,53-55,66]. Although we cannot exclude the possibility of rapid degradation by host proteins of any highly mutated copies, or occurrence of mutations outside of the *neo* gene, our results suggest that the novel ZfL2-2 and hL1 DNA copies generated in the presence of the tested APOBEC proteins are not hypermutated.

### Genome-wide analysis reveals G to A editing in lizard retrotransposons

**Figure 9:**
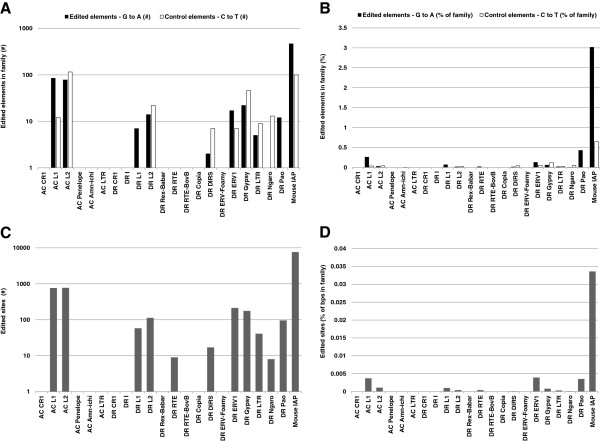
**Vertebrate genome analysis reveals G to A hypermutations in lizard retrotransposons.** LINE and LTR subfamily sequences in zebrafish (*Danio rerio* (DR)) and lizard (*Anolis carolinensis* (AC)) genomes were screened for G to A DNA editing using pairwise alignment to find clusters of G to A mismatches. C to T clusters were used as a negative control, while mouse IAP retrotransposons, where editing has previously been detected [58], served as a positive control. Editing rates are summed and presented per family. **(A)** The number of edited retrotransposons with G to A or C to T clusters in each family. **(C)** The number of edited sites in each family. Panel **(B)** presents the data from **(A)**, normalized by the number of sequences in each retrotransposon family. Panel **(D)** presents the data in **(C)**, normalized by the number of base pairs in each family. Results were produced with the P-value and threshold parameters set to 10^–8^ and 8, respectively.

Long clusters of G to A DNA mutations, a hallmark of A3 protein activity, are present in mammalian LTR and non-LTR retrotransposons [50,57,58,72-74]. Given the observed potent anti-retroelement (Figure 3) and CDA activity (Figure 4, [24]) of the lizard A1 protein, we next assessed whether G to A DNA editing is present in non-mammalian genomes, using the *in silico* method described by Carmi et al. [58]. Retrotransposon classes from zebrafish (*Danio rerio* (DR)), green anole lizard (*Anolis carolinensis* (AC)) and mouse genomes were screened. Pairwise alignments were generated between retrotransposons and clusters of G to A mutations in the retrotransposon positive strand indicated editing by AID/APOBEC proteins, while clusters of C to T mutations served as controls [58]. A significant level of DNA editing was confirmed in mouse IAP retrotransposons (Figure 9, Additional file 1), as expected for this A3 protein encoding genome, and as observed previously [58]. Importantly, a significant number of G to A edited elements was found in lizard L1 retrotransposons (85/32872 elements; ~0.26%; 7.5-fold greater than C to T control), but not in lizard L2, CR1 and LTR elements. On the other hand, a very weak and possibly negligible editing signal was observed in zebrafish retrotransposons (Figure 9, Additional file 1: Tables S2 and S3). Altogether, our genome-wide analyses provide the first evidence of G to A editing in a non-mammalian genome.

## Discussion

Our genome analysis provides the first evidence of G to A editing of retrotransposon DNA in a non-mammalian vertebrate genome, thus reflecting the presence of editing competent AID/APOBEC proteins (or other deaminases). Importantly, we report for the first time a potent anti-retroelement activity of a reptilian A1 protein, which indicates that this host-defense activity of APOBEC proteins originates in amniotes. The DNA mutator and anti-retroelement activity of reptilian A1 proteins suggests that they indeed might have been responsible for this editing. Moreover, we demonstrate that human A3 proteins inhibit both L1 and L2 retrotransposons in a sequence-independent manner. In contrast, lizard and human A1 proteins blocked only ORF1p encoding LINE elements, revealing a differential mechanism of action of A1 and A3 proteins against LINE retrotransposition.

### The anti-retroelement activity of A1 proteins predates mammals

AID/APOBEC proteins are present in all vertebrates (summary in Figure 1), but their distribution, evolution and functions differ. Fish genomes encode AID and A2 members of the APOBEC family, but lack the A1 and A3 genes [23]. However, our genomic analyses did not reveal any DNA editing in zebrafish LINE elements (Figure 9). In accordance, our data demonstrate that zebrafish AID/APOBEC proteins have a negligible effect on ZfL2-1, ZfL2-2 and hL1 retrotransposition (Additional file 1: Figure S2). Along with the low DNA mutator activity of the zebrafish A2b protein, these results collectively indicate that the anti-LINE activity of APOBEC proteins evolved later during vertebrate evolution.

In reptiles and birds, A1 homologs have been discovered [24]. Here, we confirm previous observations [24] that the anole lizard A1a protein is a potent DNA mutator (Figure 4) and show that it inhibits hL1 and ZfL2-1 retrotransposition (Figure 3), which suggests that the APOBEC anti-retroelement defense function exists in reptiles. The turnover of retrotransposons in reptiles is very rapid and only a few copies of each retrotransposon family are present in their genomes, suggesting that host factors efficiently prevent their fixation [25]. The identification of the latter awaits further studies, but our results suggest that the lizard A1 protein could be one of the anti-retroelement proteins in reptiles. The lizard A1 protein is expressed in the gastrointestinal tract and it lacks the C-terminal region present in mammalian A1 proteins essential for their apoB RNA editing function [24,41]. It has thus been suggested that the DNA editing function of A1 proteins predates their ability to modify RNA in mammals [24]. Therefore, the DNA mutator and anti-retroelement activity of the lizard A1 protein, along with the G to A DNA hypermutations detected in the lizard genome, suggest that the anti-retroelement activity of A1 proteins emerged in amniotes.

### A3 and A1 proteins differentially inhibit LINE retrotransposition

Our comparative analyses of zebrafish, lizard and human AID/APOBEC-induced inhibition of hL1, ZfL2-1 and ZfL2-2 retrotransposons demonstrate that of the human A3 proteins, A3A and A3B most potently inhibit fish L2 retrotransposition (Table 1), with rates and a mechanism (Figures 5, 6, 7 and 8) resembling their inhibition of hL1 [49,51,53,65,66,69]. In fact, several similarities between A3-induced inhibition of L1 and L2 retrotransposition exist: (1) the A3A-induced inhibition requires a conserved active site (Figure 6); (2) the A3B-induced inhibition is likely dependent on enzymatic activity and the integrity of at least one domain is needed (Figure 7); (3) A3A and A3B proteins decrease the level of novel retrotransposon DNA copies (Figure 8A); (4) in novel retrotransposon DNA copies, no obvious hyperediting was found (Additional file 1: Figure S7). This suggests that the A3-induced restriction of L1 and L2 retrotransposons in HeLa cells occurs in an editing-independent manner, regardless of the presence or absence of ORF1p in the elements, and includes a decrease in the level of novel retrotransposon DNA. In contrast, the effect of both lizard and human A1 proteins on the level of novel retrotransposon DNA was negligible (Figure 8B). Additionally, they only blocked the ORF1p encoding hL1 and ZfL2-1 retroelements, but not the ZfL2-2 element (Figure 3), which lacks ORF1p [11,60], suggesting that the inhibition of LINE retrotransposons by A1 proteins is ORF1p-dependent. ORF1p is an RNA binding protein that forms ribonucleoprotein particles (RNPs) [3-7,75] and has been proposed to stabilize L1 RNA [76]. It is possible that the A1 protein interferes with the retrotransposition process by affecting these RNPs. Indeed, mammalian A1 proteins have been detected in high-molecular-mass RNP complexes in 293T cells [55]. The absence of ORF1p in the ZfL2-2 retrotransposon could preclude such interactions and the ability of A1 protein to inhibit retrotransposition. The selective activity of the human and lizard A1 proteins against LINE retrotransposons suggests that the mechanism of anti-retroelement activity of A1 proteins has been conserved during vertebrate evolution. In summary, our results clearly show that the mechanism of LINE inhibition by A1 and A3 proteins is different.

**Table 1:** Cytidine deaminase activity and inhibitory potency of AID/APOBEC proteins against hL1, ZfL2-2 and ZfL2-1 retroelements

**AID/APOBEC protein family**				
**Tested protein**	**CDA in *E. coli***	**Inhibition of retrotransposition**		
		**hL1**	**ZfL2-2**	**ZfL2-1**
A3A	**+ **^1^	+++ ^*,2^	+++ ^*^	+++ ^*^
A3B	**+ **^1,3^	+++ ^*,4^	+++ ^*^	ND
A3C	**+ **^5^	++ ^*,6^	++ ^*^	ND
A3F	**+ **^7^	++ ^*,4^	++ ^*^	ND
A3D	ND, edits viral cDNA ^8^	– ^*,4^	– ^*^	ND
A3G	**+ **^5^	– ^*,4^	– ^*^	ND
A3H	**+ **^10^	– ^*,11^	– ^*^	ND
hA1	**+ **^5^	+^* ^**/ ++**^12^	– ^*^	+ ^*^
hA2	**– **^5^	– ^*,2^	– ^*^	ND
hAID	**+ **^5^	– ^*,6^	– ^*^	ND
lizA1aF3	+ ^*,13^	+ ^*^	– ^*^	+ ^*^
lizA1aF8	+ ^*,13^	+ ^*^	– ^*^	+ ^*^
zfAID	+ ^*,14^	– ^*^ / **+**^14^	– ^*^	– ^*^
zfA2a	– ^*^	– ^*^	– ^*^	– ^*^
zfA2b	+ ^*^	– ^*^	– ^*^	– ^*^

The basic structural differences of the tested retrotransposons are schematically represented. *, results of this study; +++, 70% inhibition; ++, 50% inhibition; +, 30% inhibition; ND, not determined; 1, [51]; 2, [49]; 3, [71]; 4, [65]; 5, [62]; 6, [66]; 7, [77]; 8, [78]; 9, [50]; 10, [31]; 11, [53]; 12, [55]; 13, [24]; 14, [56].

### G to A DNA editing identified in a non-mammalian vertebrate genome

Whole genome analyses have previously provided evidence of DNA editing in LTR and non-LTR retrotransposons in human and mouse genomes [58]. In this study, a clear G to A editing signal was identified in L1, but not in L2 retrotransposons of the lizard genome (Figure 9), despite their high diversity and low fixation rate [19,20,25]. These findings suggest that some retrotransposons are/were preferentially edited over others. Interestingly, both lizard and human A1 proteins interfered with hL1 and ZfL2-1, but not ZfL2-2, retrotransposons *in vitro* (Figure 3). On the other hand, the rates of editing observed in L1 and L2 retrotransposons in the vertebrate genomes analyzed were low. This is in accordance with the fact that despite the significant inhibition of retrotransposition (Figures 3 and 5), no lizard or human APOBEC-induced editing was detected in the novel hL1 DNA (Additional file 1: Figure S7), as shown previously for human APOBECs [49,51,53-55,66], nor in ZfL2-2 DNA copies in our *ex vivo* assays (Additional file 1: Figure S7). Altogether, our results suggest that (1) G to A DNA editing of retrotransposons predates mammals, (2) that retrotransposons were differentially edited during evolution, and (3), that, based on the low editing rates of the vertebrate LINE retrotransposons and absence of hypermutations in the sequenced LINE copies, AID/APOBEC proteins may have impacted the evolutionary dynamics of LINE retrotransposons also in an editing-independent manner.

## Conclusion

Altogether, the results of our *ex vivo* experiments, summarized in Table 1, along with our genomic analyses, indicate that the impact of AID/APOBEC proteins on the evolution of retrotransposons is broader than previously thought. The DNA editing detected in the lizard genome, the DNA mutator and the anti-retroelement activity of the lizard A1 protein shown here, suggest that the emergence of A1 proteins in reptiles is associated with the development of anti-retroelement activity in amniotes. Moreover, we observed that human and lizard A1 proteins restricted only ORF1p encoding LINE elements, while the presence of ORF1p was not necessary for anti-LINE activity of A3 proteins. Additionally, A3, but not A1, proteins potently restricted LINE retrotransposition by decreasing the level of novel retrotransposon DNA. The differential mechanism of LINE inhibition by A1 and A3 proteins, along with the differential editing of retroelements detected in vertebrate genomes, suggest that the early evolutionary appearance of APOBEC proteins may have importantly contributed to the different levels of expansion of LINE elements during evolution and enabled the vertebrates to cope with the harmful effects of retroelements.

## Methods

### Plasmids

Zebrafish AID, A2a and A2b encoding fragments were PCR amplified from previously described plasmids [36] using the primer pairs zAIDKpnIf/zAIDNotIr, zA2aKpnIf/zA2aNotIr and zA2bKpnIf/zA2bNotIr (Additional file 1: Table S1), and cloned into the pcDNA6.2 vector (Invitrogen). Lizard A1 variants F3 (R73K, Y116C) and F8 (N52D) coding fragments were amplified with lizA1HindIIIs and lizA1XhoIas primers from bacterial pTrc99A-based expression vectors that were a kind gift from prof. Conticello [24], and subcloned into the pcDNA6.2 plasmid. The AID and APOBEC bacterial expression plasmids used in the mutational assay were obtained by subcloning into the pTrc99A plasmid [63]. Plasmids encoding the full length hL1 (pJM101 [60]), UnaL2 (pI-29 [12]), ZfL2-2 (pBZ2-5) and ZfL2-1 (p77.52) [11] retrotransposons, all of them marked with the *neo* cassette, were described previously. Plasmids encoding retrotranspositionally defective retrotransposons, the ZfL2-2 and ZfL2-1 3′ UTR deletion mutants (pBB4 and p74.18) [11], the hL1 ORF1p mutant (pJM111) and the ORF2p mutant (pJM105) with no reverse transcription activity [60], were used as negative controls. All retrotransposon plasmids also carried a hygromycin resistance gene that enabled selection of successfully transfected cells. Vectors expressing mouse A3 and human A1, A2, AID, A3C, A3F, A3G, A3D, A3H, A3H-L, A3A and A3B proteins were prepared from previously described plasmids [51,78-81]. The plasmids encoding the A3A E72A, CC101,106AA, ECC72,101,106AAA, F75L and F95L mutants were prepared using the QuickChange II Site-Directed Mutagenesis kit (Stratagene) and the primers A3A.E72Af, A3A.E72Ar, A3A.CC101,106AAf and A3A.CC101,106AAr, as well as previously reported primers [68]. NA3B and CA3B mutants encoding fragments were PCR amplified from the pKA3B plasmid [51,61] using the primer pairs NA3B.HindIIIs/NA3B.XhoIas and CA3B.HindIIIs/CA3B.XhoIas, respectively, and cloned into the pcDNA6.2 plasmid. Plasmids encoding NA3B E68Q, A3B E68Q, A3B E255Q and A3B EE68, 255QQ mutants were prepared by site directed mutagenesis using the primers A3B.E68Qs, A3B.E68Qas, A3B.E255Qs and A3B.E255Qas. All plasmids were verified by sequencing. All primer sequences can be found in Additional file 1: Table S1.

### *E. coli* mutation assay

The mutation assay was performed as described previously [24,63,64] using the *E. coli* strain KL16 (Hfr (PO-45) *relA1 spoT1 thi-1*) and its UDG-deficient derivative (BW310). Briefly, KL16 and BW310 cells were transformed with AID/APOBEC expression constructs or vector alone. Individual colonies were picked and grown to saturation in a liquid LB medium containing 100 μg/ml ampicillin and 1 mM isopropyl 1-thio-β-D-galactopyranoside (IPTG), with the exception of control experiments, in which the IPTG inducer was omitted. An aliquot of saturated culture was plated onto LB medium containing 100 μg/ml rifampicin and 100 μg/ml ampicillin to select Rif^R^ colonies, and an appropriate dilution was plated onto LB medium containing 100 μg/ml ampicillin for viable cell count. All plates were incubated overnight at 37°C to allow colony formation. Mutation frequencies were measured by determining the median number of colony forming cells that survived selection per 10^9^ viable cells plated. Each median was determined from 8–16 independent cultures. IPTG induced expression of AID and APOBEC proteins conferred no obvious defect in cell growth or viability.

### Immunoblotting

Human embryonic kidney 293T cells were transfected with 1 μg of APOBEC encoding plasmid. 48 h after transfection, cells were lysed in RIPA lysis buffer (1% NP-40, 0.1% SDS, 150 mM NaCl, 50 mM Tris–HCl, pH 7.6) with Protease Inhibitor Cocktail (Sigma) and incubated for 40 min at 4°C. After separation of cell lysates by SDS-PAGE, western blotting using α-HA (Sigma), α-Myc, α-V5 and α-GAPDH monoclonal antibodies (Invitrogen) and the appropriate HRP-conjugated secondary antibodies was performed. Detection with ECL reagent (Pierce) followed.

### Retrotransposition and toxicity assays

The effect of AID/APOBEC proteins on retrotransposition and their possible cytotoxic effect were measured as previously described [51,53,60,61]. Briefly, HeLa cells were seeded in 6-well dishes at a density of 2 × 10^5^ cells per well, left to attach for 24 h, and co-transfected using 6 μl of Fugene® 6 Transfection Reagent (Roche), with 1 μg of a retrotransposon encoding plasmid (pJM101, pBZ2-5, or pI-29), or, for cytotoxicity testing, with the *neo*-expressing pcDNA3.1 plasmid (Invitrogen) instead, and with 1 μg of the respective AID/APOBEC protein encoding plasmid, or an empty vector (pcDNA6.2). In the case of the ZfL2-1 retrotransposon, a total of 1.6 × 10^6^ cells per 10 cm plate were seeded and 24 h later co-transfected with 2.5 μg of each retrotransposon and AID/APOBEC encoding plasmid. 2 days post-transfection, cells were reseeded on 10 cm dishes, incubated for 24 h, and incubated with G418 (500 μg/ml) for the next 12 days. Resistant colonies were fixed with methanol, stained with trypan blue and counted. Assays with plasmids encoding the retrotranspositionally defective ZfL2-2 (pBB4) and ZfL2-1 (p74.18) [11] or hL1 (pJM111 and pJM105) [60] retrotransposons and plasmids encoding AID/APOBEC proteins were performed as negative controls. In addition, cells were transfected only with AID/APOBEC plasmids. In both cases, no resistant colonies were formed (data not shown). The retrotransposition rate in the presence of the empty pcDNA6.2 vector was set to 100%. Retrotransposition efficiency values are means ± SD from at least three independent experiments performed in triplicates. Statistical significance between two groups was calculated by performing the Student’s *t*-test. A value of P < 0.05 was considered to be of statistical significance.

### DNA isolation with sequencing and qPCR analyses of novel LINE DNA and plasmid DNA levels

For sequencing and analysis of novel retrotransposon DNA HeLa cells were co-transfected with 1 μg of APOBEC and 1 μg of retrotransposon plasmids as described above, and transfectants containing the retrotransposon plasmid were selected with hygromycin (200 μg/ml) for 4 days. Total DNA (both chromosomal and episomal) was isolated using the DNeasy Blood & Tissue Kit (Qiagen) and contaminating RNA was degraded with RNase A from bovine pancreas (Roche).

To analyze the novel retrotransposon DNA for mutations (Additional file 1: Figure S7), 600 ng of DNA per sample in 20 μl reactions was subjected to Platinum Taq DNA Polymerase High Fidelity (Invitrogen) PCR amplification of the *neo* gene with primers neo437S and neo1808AS [60]. The integrity of the isolated DNA was verified by amplification of the single copy *PGK* gene. After gel electrophoresis, the intronless *neo* PCR products were isolated, purified and cloned into pCR4 TOPO TA cloning vectors (Invitrogen). Plasmids were isolated from at least 5 individual colonies and the *neo* insert was sequenced using T7 primers. Nucleotide sequences of independent clones were aligned and analyzed using the Hypermut software [82].

To determine the relative abundance of novel ZfL2-2 and hL1 copies in total DNA, quantitative PCR analysis was performed. A 117 bp fragment of the spliced and reversely transcribed *neo* gene was amplified with an internal sense primer (Neo210s) and an exon-exon junction spanning antisense primer (Neo1228as), which specifically recognizes the spliced *neo* cassette. Primers targeting two single-copy genes, superoxide dismutase (*SOD2*) and haemoglobin beta (*HBB*) [83], were used for normalization of spliced *neo* DNA levels. All reaction mixtures contained 500 nM of each primer and 2.5 ng of DNA in a final volume of 10 μl. qPCR was carried out on a LightCycler Nano system (Roche) using FastStart Essential DNA Green Master (Roche) chemistry at the following cycle conditions: one cycle at 95°C for 600 s; 45 cycles at 95°C for 15 s, 60°C for 12 s, 72°C for 15 s, and one cycle at 72°C for 120 s. The specificity of each primer pair was confirmed by melting curve analysis and gel electrophoresis following each run (Additional file 1: Figure S8). A calibrator DNA sample, isolated from HeLa cells co-transfected with the retrotransposon and an empty plasmid was used for determination of PCR reaction efficiency and for inter-run variability correction. An established comparative Cq method with efficiency correction, error propagation and calibrator normalization was used for relative quantification using both reference genes [84]. Results are presented relative to the *neo* frequency obtained with the DNA sample isolated from cells co-transfected with an empty vector and the retrotransposon plasmid.

To test the effect of APOBEC proteins on plasmid DNA, cells were co-transfected as described above, either with the ZfL2-2 encoding plasmid or an empty pcDNA6.2 vector, and the APOBEC encoding plasmid. 48 h after transfection, total DNA was isolated, and the effect of APOBEC proteins on plasmid DNA was analyzed with qPCR. Levels of the ZfL2-2 retrotransposon encoding plasmid were measured by amplification of a 109 bp fragment using the primer pair Neo673F/Int738R, while pcDNA6.2 plasmid level was detected with the primer pair Blas149F/Blas248R amplifying a 100 bp long fragment within the blasticidin resistance gene. Plasmid levels were normalized to genomic DNA levels by measuring the HBB and SOD2 gene levels, and the reaction conditions were essentially the same as described above, except that 0.025 ng of total DNA was used per reaction. All primer sequences are listed in Additional file 1: Table S1.

### RNA isolation, reverse transcription and qPCR analysis

RNA was isolated 2 days post-transfection using the RNeasy Plus Mini kit (Qiagen) and contaminating DNA was degraded using the DNA-free Kit (Ambion). Reverse transcription was performed in 10 μl reactions with 1.25 μg of each RNA sample using the Superscript Vilo cDNA Synthesis Kit (Invitrogen). Negative controls without template or reverse transcriptase were included in all assays. Resulting cDNA was diluted 4000-fold and subjected to qPCR analyses with primer pair Neo673F/Int738R (Additional file 1: Table S1). The GAPDH reference gene was amplified with the GAPDHf/GAPDHr primer pair. The reaction conditions were the same as for the DNA qPCR analysis described above.

### Genome-wide analysis of G to A editing in vertebrate genomes

All genomic repeats in the LINE and LTR classes were downloaded from the UCSC table browser [85] ‘rmsk’ table via the “Galaxy” online genomics tool (http://main.g2.bx.psu.edu/) [86-88] for mouse, lizard and zebrafish genomes (mm9, anoCar2 and danRer7 genomic builds, respectively). Additionally, we downloaded the latter 2 genomes from the UCSC genome browser’s [89] FTP site (ftp://hgdownload.cse.ucsc.edu/) and ran RepeatMasker (http://www.repeatmasker.org) with libraries of repeats of interest that were not present in the ‘rmsk’ table (see Additional file 2). The genomic sequences respective to the coordinates identified by RepeatMasker were extracted from the genomes and united with the ‘rmsk’ table data. Retrotransposon sequences of each genome were sorted by subfamilies. The subfamily annotation was assigned by RepeatMasker and was derived from Repbase Update’s [90] three levels of classification that we addressed as ‘Class’ , ‘Family’ and ‘Subfamily’ (e.g. LINE class, L1 family, L1_AC_11 subfamily (Additional file 1: Tables S2 and S3)). The DNA editing detection was performed using the method described by Carmi et al. [58]. The method contains two steps: generating pairwise alignments of all sequences of the same subfamily using BLAST [91] and screening the alignments for DNA editing with PERL scripts. Briefly, pairwise alignments between similar sequences in LINE and LTR classes of retrotransposons from the zebrafish, lizard and mouse genomes were generated using BLAST [91]. The alignments were then screened for one or more clusters of consecutive G to A mutations, and the resulting editing rates compared to those previously detected in the human (L1 and SVA) and mouse genomes (L1 and IAP) [58]. Based on the strand specificity of DNA editing, complementary event of C to T mutations was used as control. The goal was to detect pairs of ‘parent’ and ‘child’ sequences, where the child has a cluster of G to A mutations in comparison to the parent, denoting that its DNA was edited by APOBEC proteins during retrotransposition. The high similarity we demanded between these two sequences, enforced by the BLAST parameters (E-value of 10^-50^) and our algorithm, provides significant support that the child sequence indeed evolved from the parent sequence. The DNA editing finding algorithm searches for a pair of sequences with a series, or cluster, of G to A mismatches. To reduce the false-positive rate, we do not allow any other type of mismatch in the G to A cluster. The cluster’s significance is measured by two values: (i) ‘Threshold’ - the number of G to A mismatches in the cluster, (ii) ‘P-value’ - the P-value calculated by the Binomial probability of arbitrarily getting such a cluster of transitions in the given alignment. The probability of a single transition is calculated by the number of T to C and C to T transitions in the alignment. A3 proteins introduce G to A mutations in the retrotransposon’s positive strand as defined by its ORF. For control, we exploited this strand specificity by using the G to A ‘editing’ of the minus strand to test the significance of our results. Our data contains only the retrotransposon positive strands, thus, this event is represented as C to T ‘editing’ in our data. We present the same parameters used by Carmi et al. [58], which contain a set of parameters with extremely high significance (P-value = 1e-13 and Threshold = 9) and a set of slightly relaxed parameters (P-value = 1e-8 and Threshold = 8). The data in the manuscript was generated using the latter (for details, see also Additional files 1 and 2). We applied the method to all LINE and LTR repeats in the zebrafish and lizard genomes and compared the results to significantly edited families in human and mouse genomes.

## Abbreviations

LTR: Long terminal repeat; LINE: Long interspersed nuclear element; L1: LINE1; L2: LINE2; UTR: Untranslated region; ORF1p and ORF2p: Open reading frame 1 and 2 retrotransposon proteins; RNPs: Ribonucleoprotein particles; CDA: Cytidine deaminase activity; AID: Activation-induced deaminase; A1: APOBEC1; A2: APOBEC2; A3: APOBEC3; A4: APOBEC4; A5: APOBEC5; hL1: human L1; ZfL2-2: zebrafish L2-2; IAP: Intracisternal A-particle; ZfL2-1: Zebrafish L2-1; UnaL2: eel L2; neo: Neomycin resistance gene; qPCR: Quantitative PCR.

## Competing interests

The authors declare that they have no competing interests.

## Authors’ contributions

NLi carried out most of the *ex vivo* experiments. MB performed the *E. coli* mutation assay. BAK and EYL carried out the *in silico* genomic analysis. NLi, NLo, TP, MB, BAK and EYL interpreted the results and wrote the manuscript. NLo conceived and coordinated the study. All authors participated in drafting the manuscript and read and approved the final manuscript.

## Supplementary Material

Additional file 1 **A PDF document containing supplemental data for this paper.** Included are eight supplemental figures (Additional file 1: Figures S1–S8), three supplemental tables (Additional file 1: Tables S1–S3) and details about the *in silico* genomic analysis with the information about dataset construction, analysis of results and description of the Additional file 2 spreadsheet. Click here for file

Additional file 2 **An Excel spreadsheet (XLS) that corresponds to the data in Additional file 1, Section 3, Coordinates of editing detection pairs.** The spreadsheet contains all the coordinates of editing pairs used in our *in silico* genomic analyses. Click here for file
