# Supplementary material for: Differential inhibition of LINE1 and LINE2 retrotransposition by vertebrate AID/APOBEC proteins
Source: Retrovirology. 2013 Dec 17;10:156. doi: 10.1186/1742-4690-10-156 (PMC3880073; doi:10.1186/1742-4690-10-156)
Supplement: Additional file 1 — A PDF document containing supplemental data for this paper. Included are eight supplemental figures (Additional file 1: Figures S1–S8), three supplemental tables (Additional file 1: Tables S1–S3) and details about the in silico genomic analysis with the information about dataset construction, analysis of results and description of the Additional file 2 spreadsheet. [file 1742-4690-10-156-S1.pdf]

Differential inhibition of LINE1 and LINE2 retrotransposition by vertebrate AID/APOBEC proteins

Nataša Lindič, Maruška Budič, Toni Petan, Binyamin A. Knisbacher, Erez Y. Levanon, Nika Lovšin

Additional file 1

Supplementary materials include three tables (S1–3) and eight figures (S1–8) and consist of two parts:

Part (1): *Ex vivo* experiments

Figure S1. Evaluation of cytotoxic effects of the tested AID/APOBEC proteins.
Figure S2. The effect of zebrafish AID/APOBEC proteins on the retrotransposition of ZfL2-2, hL1 and ZfL2-1 retrotransposons in HeLa cells is negligible.
Figure S3. Representative experimental results of the *neo*-based retrotransposition assay.
Figure S4. Human A2, AID, A3D and A3H-L proteins do not inhibit ZfL2-2 retrotransposition.
Figure S5. APOBEC proteins do not significantly alter the levels of transfected plasmid DNA.
Figure S6. APOBEC proteins do not affect the level of ZfL2-2 or hL1 RNA.
Figure S7. Sequence analyses of novel ZfL2-2 and hL1 DNA copies.
Table S1. List of primers used in this study.
Figure S8. Confirmation of qPCR amplification specificity.

Part (1): *In silico* analysis of DNA editing of genomic retrotransposons

Table S2. G to A editing results of genomic analyses performed with 'low confidence' parameters (P-value = 10^{-8}, Threshold = 8).
Table S3. G to A editing results of genomic analyses performed with 'high confidence' parameters (P-value = 10^{-13}, Threshold = 9).

Part (1): *Ex vivo* experiments

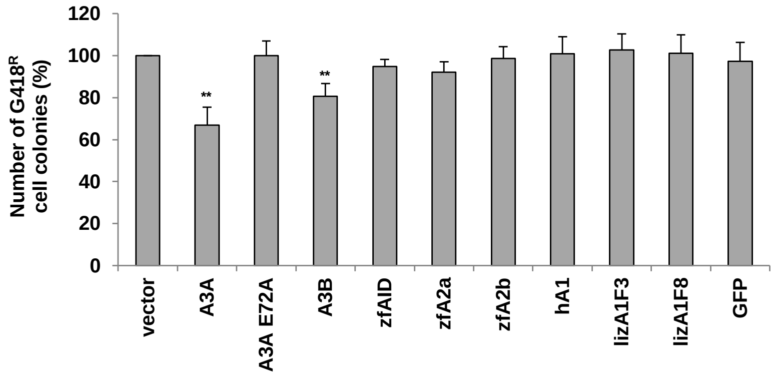

Figure S1. Evaluation of cytotoxic effects of the tested AID/APOBEC proteins.
HeLa cells were co-transfected with 1 µg of *neo*-resistance encoding pcDNA3.1 plasmid and 1 µg of AID/APOBEC protein encoding plasmid or an empty parental pcDNA6.2 vector and a GFP encoding plasmid as control. After 12 days of G418 selection, neomycin resistant colonies ($G418^R$) were fixed, stained and counted, and relative cytotoxicity was calculated by setting the value for cells, co-transfected with the pcDNA3.1 plasmid and an empty pcDNA6.2 vector, as 100%. Data are the means ± standard deviations (SD) of three independent co-transfection experiments. Except for a weak cytotoxic effect of A3A and A3B proteins, the overexpression of AID/APOBEC had no considerable effect on cell viability. ***$P < 0.001$, **$P < 0.01$, *$P < 0.05$, *t*-test.

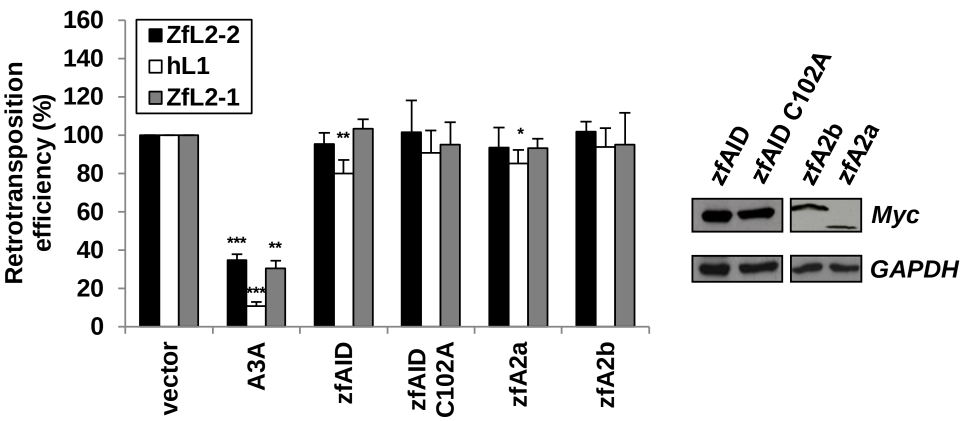

Figure S2. The effect of zebrafish AID/APOBEC proteins on the retrotransposition of ZfL2-2, hL1 and ZfL2-1 retrotransposons in HeLa cells is negligible.
Retrotransposition efficiency of zebrafish L2-2 and L2-1 (ZfL2-2 and ZfL2-1) and human L1 (hL1) retrotransposons in HeLa cells co-transfected with 1 µg of target retrotransposon plasmid and 1 µg of effector plasmids encoding human A3A, zfAID, zfA2a and zfA2b proteins, and the zfAID C102A mutant. After G418 selection, neomycin resistant colonies were fixed, stained and counted, and relative retrotransposition was calculated by setting the value for cells, co-transfected with retrotransposon plasmid and an empty pcDNA6.2 vector, as 100%. Successful expression of Myc epitope-tagged zebrafish proteins in mammalian cells was confirmed in the HEK293T model. GAPDH was used as a loading control. Data are the means ± standard deviations (SD) of at least three independent experiments. ***$P < 0.001$, **$P < 0.01$, *$P < 0.05$, *t*-test.

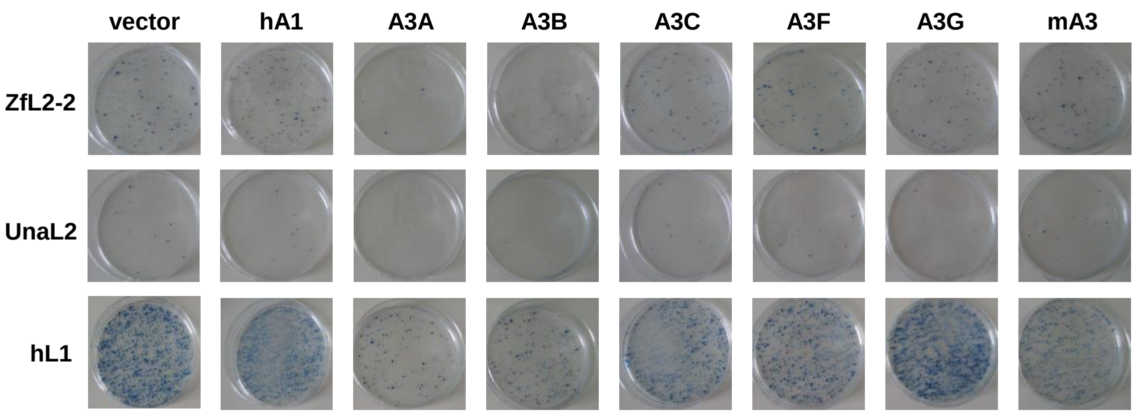

Figure S3. Representative experimental results of the *neo*-based retrotransposition assay.
HeLa cells were co-transfected with target plasmids encoding ZfL2-2, eel L2 (UnaL2) and hL1 retrotransposons, and effector plasmids coding for the human A1 (hA1) or A3 proteins, mouse A3 (mA3) protein or an empty vector (pcDNA6.2). 3 days after transfection cells were subjected to G418 selection for 12 days. Experiments representative of the data compiled in Figure 5A show neomycin resistant colonies obtained after 12 days of selection.

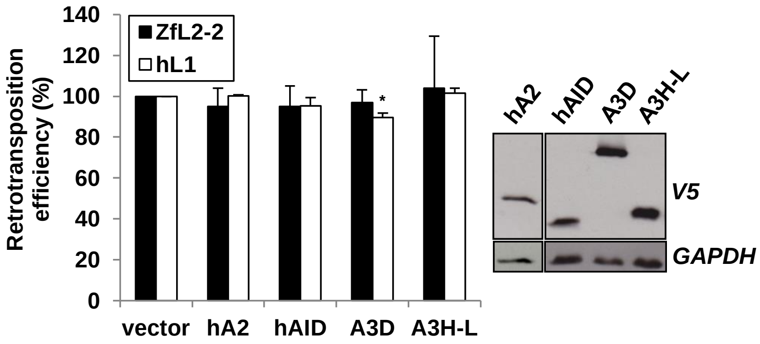

Figure S4. Human A2, AID, A3D and A3H-L proteins do not inhibit ZfL2-2 retrotransposition. Retrotransposition efficiency of ZfL2-2 and hL1 retrotransposons in HeLa cells co-transfected with 1 µg of retrotransposon plasmid and 1 µg of plasmid encoding human A2 (hA2), AID (hAID), A3D and A3H-L (haplotype I) proteins was determined by counting fixed and stained neomycin resistant colonies, formed after G418 selection, and calculated by setting the value for cells, co-transfected with retrotransposon plasmid and an empty vector, to 100%. Successful expression of V5 epitope-tagged hA2, hAID, A3D and A3H-L proteins was confirmed in the HEK293T model. Due to the lack of the premature termination codon [80], we could only detect the expression of the full-length mutant hA3H-L protein, while the wild-type hA3H protein was undetectable. GAPDH was used as a loading control. Data are the means ± standard deviations (SD) of at least three independent experiments. ***P < 0.001, **P < 0.01, *P < 0.05, *t*-test.

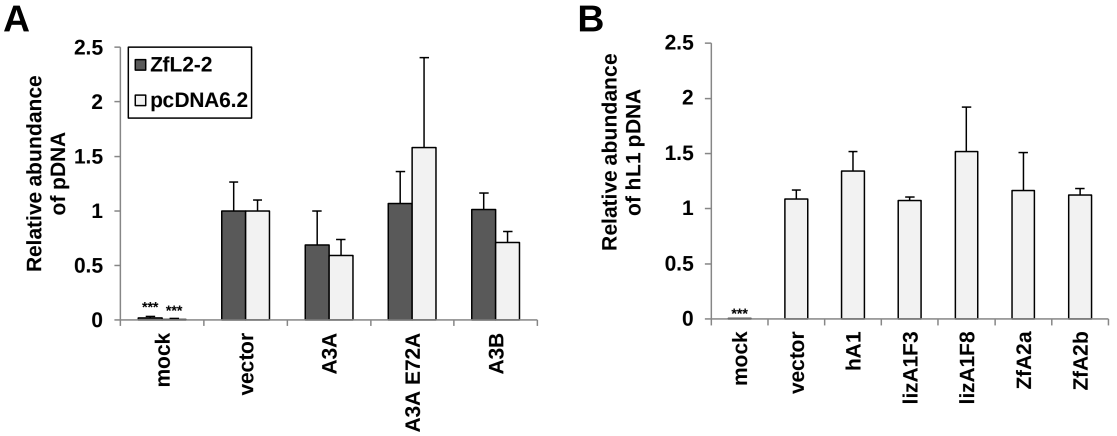

Figure S5. APOBEC proteins do not significantly alter the levels of transfected plasmid DNA. To test the effect of APOBEC proteins on plasmid DNA, cells were co-transfected with 1 µg of the indicated APOBEC encoding plasmid and either with 1 µg of the ZfL2-2 encoding plasmid (pBZ2-5) or 1 µg of the empty pcDNA6.2 vector (**A**), or hL1 encoding plasmid (**B**). 48 h after transfection, total DNA was isolated, and the effect of APOBEC proteins on plasmid DNA was estimated by qPCR. A 109 bp fragment spanning the boundary between the intron and the neomycin gene was amplified to detect the ZfL2-2 encoding plasmid, and the level of pcDNA6.2 plasmid was determined by amplifying a 100 bp long sequence within the blasticidin resistance gene. Plasmid levels were normalized to HBB and SOD2 gene levels. There were no statistically significant effects of APOBEC proteins on the level of plasmid DNA ($P > 0.05$, *t*-test). Values obtained with DNA from cells transfected with an empty vector were set as 1. Histogram bars represent the means ± SD of two independent experiments. Mock controls represent untransfected cells. ***$P < 0.001$, **$P < 0.01$, *$P < 0.05$, *t*-test.

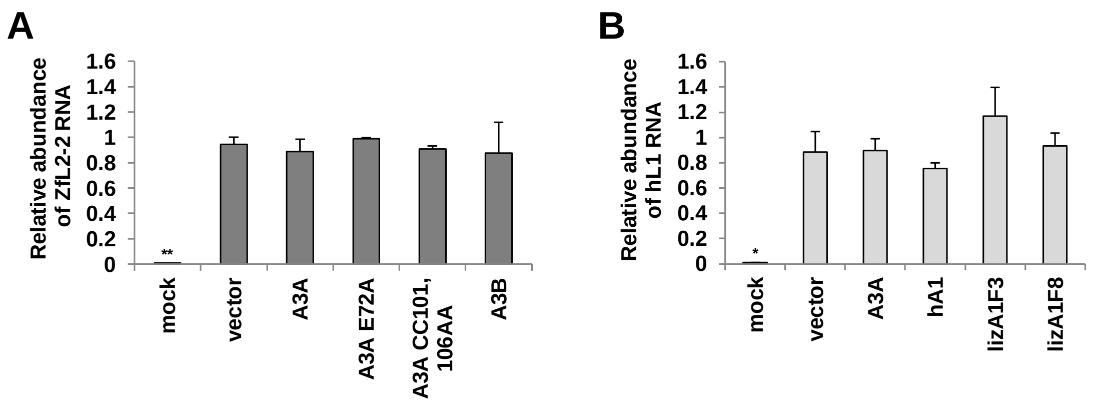

Figure S6. APOBEC proteins do not affect the level of ZfL2-2 or hL1 RNA.
qPCR analysis of intronless ZfL2-2 and hL1 *neo* transcripts in the presence of human A3A and A3B proteins or their mutants (**A**), or human A3A and A1 (hA1) and lizard A1 proteins (**B**). HeLa cells were co-transfected with 1 μg of the target plasmids encoding ZfL2-2 or hL1 retrotransposon and 1 μg of the APOBEC effector plasmid. Two days after transfection, RNA was isolated, retrotranscribed into cDNA, and quantified by qPCR. The relative levels of ZfL2-2 and hL1 cDNA were normalized to those obtained after amplification of GAPDH cDNA. Values obtained with RNA from cells transfected with an empty vector were set as 1. There were no statistically significant changes ($P > 0.05$, *t*-test) in the level of RNA caused by APOBEC proteins. Histogram bars represent the means ± SD of two independent experiments. Mock controls represent untransfected cells. ***$P < 0.001$, **$P < 0.01$, *$P < 0.05$, *t*-test.

A

A3A (To / From)

From \ To	A	C	G	T
A	---	0	0	1
C	0	---	0	0
G	2	1	---	0
T	1	1	2	---

N=2064

A3B (To / From)

From \ To	A	C	G	T
A	---	0	2	0
C	0	---	1	1
G	0	2	---	0
T	0		1	---

N=2740

A3C (To / From)

From \ To	A	C	G	T
A	---	0	0	1
C	0	---	0	0
G	0		---	0
T	0	0	2	---

N=2579

pcDNA6.2 (To / From)

From \ To	A	C	G	T
A	---	0	8	0
C	0	---	0	2
G	2	0	---	0
T	0	0	1	---

N=3499

A3F (To / From)

From \ To	A	C	G	T
A	---	0	0	0
C	0	---	0	0
G	0	0	---	0
T	0	0	0	---

N=2984

A3G (To / From)

From \ To	A	C	G	T
A	---	1	1	0
C	0	---	0	0
G	0	0	---	0
T	0	1	2	---

N=3350

mA3 (To / From)

From \ To	A	C	G	T
A	---	0	0	0
C	0	---	0	1
G	1	0	---	0
T	1	1	1	---

N=2981

zfA2b (To / From)

From \ To	A	C	G	T
A	---	0	0	0
C	0	---	0	0
G	5	0	---	0
T	0	0	0	---

N=2460

zfA2a (To / From)

From \ To	A	C	G	T
A	---	0	19	4
C	11	---	15	4
G	7	8	---	6
T	2	12	8	---

N=1936

zfAID (To / From)

From \ To	A	C	G	T
A	---	0	0	0
C	0	---	0	0
G	5	0	---	5
T	0	0	0	---

N=2460

hA2 (To / From)

From \ To	A	C	G	T
A	---	0	0	0
C	0	---	0	0
G	0	0	---	0
T	0	0	5	---

N=2460

hA1 (To / From)

From \ To	A	C	G	T
A	---	0	10	0
C	5	---	0	3
G	5	0	---	5
T	0	0	0	---

N=2445

lizA1F3 (To / From)

From \ To	A	C	G	T
A	---	0	12	2
C	5	---	2	7
G	3	4	---	6
T	1	6	4	---

N=2579

lizA1F8 (To / From)

From \ To	A	C	G	T
A	---	0	13	2
C	5	---	2	7
G	3	4	---	6
T	0	0	1	---

N=2348

pcDNA6.2 (To / From)

From \ To	A	C	G	T
A	---	0	0	0
C	0	---	0	0
G	5	0	---	5
T	0	0	0	---

N=1968

B

lizA1F3 (To / From)

From \ To	A	C	G	T
A	---	0	1	0
C	0	---	0	1
G	0	0	---	0
T	0	0	0	---

N=2445

lizA1F8 (To / From)

From \ To	A	C	G	T
A	---	0	19	4
C	7	---	7	9
G	5	7	---	5
T	4	13	5	---

N=1890

pcDNA6.2 (To / From)

From \ To	A	C	G	T
A	---	0	11	0
C	5	---	0	0
G	5	0	---	5
T	0	0	0	---

N=2445

Figure S7. Sequence analyses of novel ZfL2-2 and hL1 DNA copies.
Nucleotide substitution preferences for novel retrotransposon DNA copies in the presence of the indicated AID/APOBEC protein were examined. HeLa cells were co-transfected with target plasmids encoding the ZfL2-2 (**A**) or hL1 (**B**) retrotransposons and effector plasmids encoding the indicated AID/APOBEC protein or the empty vector (pcDNA6.2). 3 days after transfection, cells were subjected to hygromycin selection for 4 days, total cellular DNA was isolated, and PCR analysis performed. The spliced 487 bp *neo* fragments were cloned into the pCR4 TOPO TA cloning vector and at least 5 independent clones were sequenced. Mutation analysis was performed using the Hypermut software [81]. N, total number of bases.

Table S1. List of primers used in this study.

Primer name	Sequence (5' to 3')
zA2b KpnIf	ACGTGGTACCATGGCAGACAAAAAGGACAGC
zA2b NotIr	ACGTGCGGCCGCCCTTTAAAATATCCTGCAATC
zAID KpnIf	ACGTGGTACCATGATCTGCAAGCTGGACAGTGTGCTC
zAID NotIr	GCGGCCGCCTAACCCAAGAAGAGCAAAAACATCCCT
zA2a KpnIf	ACGTGGTACCATGGCCGATAGAAAGGGCAGC
zA2a NotIr	GCGGCCGCCCTGCAGGATGTCGGCCAG
A3A.E72Af	GGCCGCCATGCGGCGCTGCGCTTCTTG
A3A.E72Ar	CAAGAAGCGCAGCGCCGCATGGCGGCC
A3A.CC101.106AAf	CCTGGAGCCCCGCCTTCTCCTGGGGCGCTGCCGGGGAAGTG
A3A.CC101.106AAr	ACTTCCCCGGCAGCGCCCCAGGAGAAGGCGGGGCTCCAGGA
A3A F75Ls [68]	GGAACCAGGTCCAATAAGCGCAGCTCCGCATGG
A3A F75Las [68]	CCATGCGGAGCTGCGCTTATTGGACCTGGTTCC
A3A F95Ls [68]	GGCTCCAGGAGATTAACCAAGTGACCCTGTAG
A3A F95Las [68]	CTACAGGGTCACTTGGTTAATCTCCTGGAGCC
NA3B.HindIIIs	GAAAGCTTATGAATCCACAGATCAGAAAT
NA3B.XhoIas	TCGAGCCAGGTATCTGAGAATCTC
CA3B.HindIIIs	GAAAGCTTATGGATCCAGACACATTCACTTTC
CA3B.XhoIas	TCGAGCGTTTCCCTGATTCTGGAGAATA
A3B.E68Qs	AAGCCTCAGTACCACGCACAAATGTGCTTCCTC
A3B.E68Qas	GAGGAAGCACATTTGTGCGTGGTACTGAGGCTT
A3B.E255Qs	GGCTTTTACGGCCGCCATGCGCAGCTGCGCTTCTTG
A3B.E255Qas	CAAGAAGCGCAGCTGCGCATGGCGGCCGTAAAAGCC
lizA1HindIIIs	GAATAAGCTTATGGAATTCGCTGCAATTC
lizA1XhoIas	GATTCTCGAGGTCCTGAAGAATTGAGTCA
neo437S [60]	GAGCCCCTGATGCTCTTCGTCC
neo1808AS [60]	CATTGAACAAGATGGATTGCACGC
PGKf	CAGTTTGGAGCTGGAAG
PGKr	TGCAAATCCAGGGTGCAGTG
Neo210s	CCCAATAGCAGCCAGTCCCTT
Neo1228as	TGAATGAGCTCTTCAGGACGAGG
Neo673F	CTTCAGTGACAACGTCGAGCAC
Int782R	CAGTGCTGAAACATCTCCTGGAC
Blas149F	GGGACCTTGTGCAGAACTCGT
Blas248R	GATGCCCCTGTTCTCATTTCCG
SOD2 F [82]	GGAGAAGCTGACGGCTGC
SOD2 R [82]	CCTTATTGAAACCAAGCCAACC
HBB F [82]	GTGAAGGCTCATGGCAAGAAAG
HBB R [82]	CAGCTCACTCAGTGTGGCAAAG
GAPDHf	CAACGGATTTGGTCGTATTGG
GAPDHr	GCAACAATATCCACTTTACCAGAGTTAA

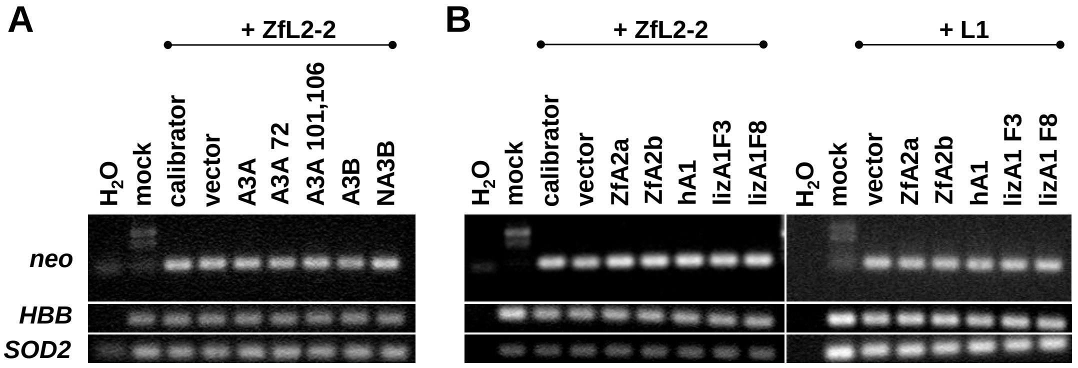

Figure S8. Confirmation of qPCR amplification specificity.
Total cellular DNA was subjected to qPCR after extraction from hygromycin-selected HeLa cells co-transfected with 1 µg of plasmids encoding ZfL2-2 or hL1 retrotransposon and 1 µg each of the indicated APOBEC effector plasmids. qPCR amplicons of spliced *neo* genes amplified with Neo210s and Neo1228as primers from the DNA samples were analysed by gel electrophoresis along with the amplicons of two single-copy genes, superoxide dismutase (*SOD2*) and haemoglobin beta (*HBB*) [82], that were used as a reference for normalization of the spliced *neo* DNA level. The corresponding final results of each qPCR run are presented in Figures 8A and B. Mock controls represent untransfected cells.

Part (2): *In silico* analysis of DNA editing of genomic retrotransposons

Section 1: Dataset construction

In addition to the UCSC table browser data, we ran the RepeatMasker for the zebrafish (danRer7) and lizard (anoCar2) genomes, using consensus sequence libraries we built, in order to retrieve retrotransposons of interest that were not present in UCSC table browser's 'rmsk' table. Many of the sequences we used were submitted to Repbase Update [84-89] after the 'rmsk' tables were generated for these genomes (the accession number of the ZfL2-2 sequence of zebrafish L2 is AB211150 [11]).

Consensus sequences contained in libraries for RepeatMasker runs (asterisks resemble the range or set of values listed in parentheses):

Lizard:

- Amn-ichi family: Amn-ichi-* (1197, 1416, 123);
- L1 family: L1_AC_* (1 to 20); L1_*_ACar (21 to 28); L1_21B_ACar; L1_24B_ACar;
- L2 family: L2_AC_* (1 to 17); L2_*_ACar (1 to 3);

Zebrafish:

- CR1 family: CR1-*_DR (L, 7, 10, 12, 15, 18, 19, 20 to 31, 37, 38, 42, 43); X*_LINE (2, 5A, 5B, 6A, 6B, 8);
- L2 family: ZfL2-2. L2-*_DR (1, 8, 31); L2-*_DRe (2, 3, 4, 5, 6, 9, 11, 13, 14, 16, 17, 32, 33, 35, 36); Eventually, we only used the ZfL2-2 sequences. The rest were already present in the dataset with other annotation (Repbase subfamily annotation was changed from CR1 to L2 prefixes after the 'rmsk' table was created in the UCSC table browser).

Data redundancy: Running the RepeatMasker created some redundancy in our data by annotating the same genomic coordinates to different repeat subfamilies (within the same family) and by annotating overlapping sequences as distinct repeats. This redundancy was removed for result-statistics analysis (Section 2A), but retained in the supplementary files (Section 3):

1. Edited sites in the genome shared by multiple elements were associated to only one of them. Next, elements that were left without any edited sites associated to them were deleted from the results.
2. To count the number of elements in each family in the initial data, we merged all overlapping coordinates and considered merged intervals as one element. These values were needed to calculate the percentage of edited elements in each family (see Table S2).

Section 2(A): Results-statistics

We used two different sets of parameters in our analysis, as in [58]:
A. 'Low confidence': P-value = 10^{-8}, Threshold = 8;
B. 'High confidence': P-value = 10^{-13}, Threshold = 9;
In the manuscript (Figure 9) we presented the first set of parameters (10^{-8}, 8) called 'low confidence' parameters after [58]. Considering that the size of the sequences population we screened was ~3.7 million, such a low P-value would enabled the detection of less than one edited sequence. Therefore, the statistical significance is still adequate. (For the same reason the C>T hypermutation found in the control is a bit surprising, future research will tell if it is an artefact or traces of some biological process).

Table S2. G to A editing results of genomic analyses performed with 'low confidence' parameters (P-value = 10^{-8}, Threshold = 8).

Hyperediting presented in the manuscript (Figure 9) was detected using pairwise alignment of retrotransposon LINE and LTR subfamily sequences in genomes of lizard (*Anolis carolinensis*) and zebrafish (*Danio rerio*). C to T clusters served as a negative and mouse IAP as a positive control [58]. Editing rates are summed and presented per family.

Organism	Class	Family	Edited elements (#)	Edited elements C to T (#)	Elements in family (#) before merge*	Elements in family (#) merged*	Edited elements (% of family)	Edited sites (#)	Edited sites C to T (#)	bps in family (#) before merge*	bps in family (#) merged*	Edited sites (% of bps)
Lizard	LINE	CR1	0	0	625	625	0	0	0	78075	78075	0
Lizard	LINE	L1	85	12	44564	32882	0.259	765	102	24448417	20948693	0.0037
Lizard	LINE	L2	78	116	440201	295347	0.026	774	1108	82415033	67838797	0.0011
Lizard	LINE	Penelope	0	0	210440	161810	0	0	0	31573500	31513175	0
Lizard	LTR	Amn-ichi	0	1	31191	23825	0	0	10	33487514	32190386	0
Lizard	LTR	LTR	0	0	25	25	0	0	0	3635	3635	0
Zebrafish	LINE	CR1	0	0	2	2	0	0	0	104	104	0
Zebrafish	LINE	I	0	0	5034	4991	0	0	0	1602323	1602036	0
Zebrafish	LINE	L1	7	0	10855	10603	0.066	58	0	5673349	5658758	0.001
Zebrafish	LINE	L2	14	22	97823	92934	0.015	113	188	26165038	25143357	0.0004
Zebrafish	LINE	Rex-Babar	0	0	13643	13040	0	0	0	4082416	4078601	0
Zebrafish	LINE	RTE	1	0	6166	5774	0.017	9	0	2178776	2175917	0.0004
Zebrafish	LINE	RTE-BovB	0	0	2455	2394	0	0	0	512109	511572	0
Zebrafish	LTR	Copia	0	0	319	255	0	0	0	174942	174902	0
Zebrafish	LTR	DIRS	2	7	22348	17584	0.011	17	66	19814985	19653906	0.0001
Zebrafish	LTR	ERV-Foamy	0	0	294	278	0	0	0	151334	151297	0
Zebrafish	LTR	ERV1	17	7	14811	13528	0.126	213	91	5415047	5409283	0.0039
Zebrafish	LTR	Gypsy	22	46	44853	39336	0.056	176	409	23250010	23231168	0.0008
Zebrafish	LTR	LTR	5	9	33169	30791	0.016	41	83	11840619	11825841	0.0003
Zebrafish	LTR	Ngaro	1	13	27018	26132	0.004	8	144	9888726	9881054	0.0001
Zebrafish	LTR	Pao	12	0	3325	2810	0.427	96	0	2777008	2775996	0.0035
Mouse	LTR	IAP	467	100	26504	15499	3.013	7637	957	22748123	22714600	0.0336

*See reason for merging in section 1 ("Data redundancy").

Table S3. G to A editing results of genomic analyses performed with 'high confidence' parameters (P-value = 10^{-13}, Threshold = 9).

Analyses were performed as described in the Table S2, except that here, 'high confidence' parameters were used.

Organism	Class	Family	Edited elements (#)	Edited elements C to T (#)	Edited sites (#)	Edited Sites C to T (#)
Lizard	LINE	CR1	0	0	0	0
Lizard	LINE	L1	16	1	165	10
Lizard	LINE	L2	26	9	272	86
Lizard	LINE	Penelope	0	0	0	0
Lizard	LTR	Amn-ichi	0	0	0	0
Lizard	LTR	LTR	0	0	0	0
Zebrafish	LINE	CR1	0	0	0	0
Zebrafish	LINE	I	0	0	0	0
Zebrafish	LINE	L1	0	0	0	0
Zebrafish	LINE	L2	0	1	0	9
Zebrafish	LINE	Rex-Babar	0	0	0	0
Zebrafish	LINE	RTE	0	0	0	0
Zebrafish	LINE	RTE-BovB	0	0	0	0
Zebrafish	LTR	Copia	0	0	0	0
Zebrafish	LTR	DIRS	0	0	0	0
Zebrafish	LTR	ERV-Foamy	0	0	0	0
Zebrafish	LTR	ERV1	10	3	143	51
Zebrafish	LTR	Gypsy	0	15	0	143
Zebrafish	LTR	LTR	0	1	0	12
Zebrafish	LTR	Ngaro	0	2	0	22
Zebrafish	LTR	Pao	0	0	0	0
Mouse	LTR	IAP	243	24	4188	251

Section 3: Coordinates of editing detection pairs

All the editing coordinates and "editing-pairs" are combined in one excel file (Additional file 2.xls) that contains the following data (worksheets):

1. Genomic coordinates of edited sites (G>A mismatches):
   A. Edited Sites AC LINEandLTR & Edited elements AC LINEandLTR
   B. Edited Sites DR LINEandLTR & Edited elements DR LINEandLTR

2. Genomic coordinates of control sites (C>T mismatches):
   A. Edited sites AC control
   B. Edited sites DR control

3. Editing pairs DR AC LINE LTR, which are the pairs of sequences whose alignments had clusters of G>A mutations. The file contains all the editing pairs for G>A editing in lizard and zebrafish LINE and LTR classes (P-value = 1e-8, Threshold = 8).

In every row, the left coordinates are the 'parent' sequence and the right coordinates belong to the 'child' sequence. Some of the edited elements were detected multiple times while aligning to different tentative parents, thus the edited sequences in every line aren't necessarily unique.

Format: Each line contains the following columns:

Assembly	Organism	Class	Family	Subfamily	Parent (Gs) sequence coordinates	Edited (As) sequence coordinates
